# Vaccine-Associated Disease Enhancement (VADE): Considerations in Postvaccination COVID-19

**DOI:** 10.1155/2021/9673453

**Published:** 2021-10-29

**Authors:** Rahajeng N. Tunjungputri, Erpryta Nurdia Tetrasiwi, Merlinda Veronica, Jacub Pandelaki, Fera Ibrahim, Erni Juwita Nelwan

**Affiliations:** ^1^Department of Internal Medicine, Faculty of Medicine Universitas Indonesia and Cipto Mangunkusumo National General Hospital, Jakarta, Indonesia; ^2^Department of Radiology, Faculty of Medicine Universitas Indonesia and Cipto Mangunkusumo National General Hospital, Jakarta, Indonesia; ^3^Microbiology Department, Faculty of Medicine, Universitas Indonesia, Jakarta, Indonesia; ^4^Division of Tropical and Infectious Diseases, Department of Internal Medicine, Faculty of Medicine Universitas Indonesia and Cipto Mangunkusumo National General Hospital, Jakarta, Indonesia; ^5^Infectious Disease and Immunology Research Center—IMERI, Faculty of Medicine, Universitas Indonesia, Jakarta, Indonesia

## Abstract

**Introduction:**

The COVID-19 pandemic has entered a new phase with the roll-out of several vaccines worldwide at an accelerated phase. The occurrence of a more severe presentation of COVID-19 after vaccination may affect policymakers' decision-making and vaccine uptake by the public. Vaccine-associated disease enhancement (VADE) is the modified presentation of infections in individuals after having received a prior vaccination. Currently, little is known about the potential of vaccine-associated disease enhancement (VADE) following COVID-19 immunization. *Case Illustration*. We herewith report two patients admitted with confirmed COVID-19 pneumonia with a history of CoronaVac vaccination. The first patient with a relatively milder course of the disease had received two doses of CoronaVac, whereas the second patient with a more progressive course of the disease received only one dose before developing symptoms and being admitted to the hospital. Our observations suggest that vaccination could act in boosting the inflammatory process and reveal the previously asymptomatic COVID-19 illness. Theoretically, vaccines could induce VADE, where only suboptimal, nonprotective titers of neutralizing antibodies were produced or proinflammatory T-helper type 2 response was induced. Secondly, enhanced respiratory disease (ERD) could manifest, where pulmonary symptoms are more severe due to peribronchial monocytic and eosinophilic infiltration. Understanding VADE is important for the decision-making by the public, clinicians, and policymakers and is warranted for successful vaccination uptake.

**Conclusion:**

We report two cases of patients developing COVID-19 shortly after CoronaVac vaccination in which VADE is likely. We recommend that current vaccination strategies consider the measurement of neutralizing antibody titer as a guide in ensuring the safest strategy for mass immunization. Studies are needed to investigate the true incidence of VADE on vaccinated individuals as well as on how to differentiate between VADE and severe manifestations of COVID-19 that are unrelated to vaccination.

## 1. Introduction

The COVID-19 pandemic has affected 109 million people, causing 2.4 million deaths worldwide to date [1]. The pandemic has now entered a new phase with the roll-out of several vaccines. The prompt announcement of the outbreak and early publication of information on the genetic sequence of the virus allowed for an unprecedentedly early initiation of COVID-19 vaccine development. In contrast, to date, although vaccine studies for some other RNA viruses such as HIV and hepatitis C are ongoing, these vaccines are not yet available for use in humans [2].

A goal of manufacturing a safe and efficacious vaccine against SARS-CoV-2 had to be achieved within a 6- to 18-month timeline, generating over 200 vaccine candidates, with 50 in human clinical trials and 18 in efficacy testing [3]. The time normally reserved for the exploration of dosing and scheduling optimization has been greatly reduced by the urgent demand for efficacious COVID-19 vaccines. The close spacing of doses has the advantage of rapid distribution of the vaccines. Ideally, the development of vaccines also prioritizes the optimal scheduling of doses that would allow for the highest immunity generated and the most durable immune response [4].

On 13 January 2021, the COVID-19 vaccine roll-out officially began in Indonesia, distributing CoronaVac, a vaccine with chemically inactivated whole-virus vaccine with alum as an adjuvant. Vaccine-associated disease enhancement (VADE) is a modified, often more severe presentation of clinical infection in individuals exposed to a pathogen after having received prior vaccination for the same pathogen. Vaccines for infection by viruses that will cause inflammatory damage are likely to result in VADE, for example, SARS-CoV and RSV [5]. Currently, little is known about the potential of VADE following COVID-19 immunization. Understanding VADE is important for the decision-making by the public, clinicians, and policymakers and is warranted for successful vaccination uptake. We herewith describe two patients with COVID-19 pneumonia who developed symptoms and presented to the emergency department each following 2 doses and 1 dose of CoronaVac vaccination.

## 2. Case Presentation

The first patient, a 24-year-old man, presented to the emergency department (ED) of Cipto Mangunkusumo Hospital (RSCM) with the chief complaint of fever which had been present for one day. Notable symptoms upon diagnosis were coughing, rhinorrhea, nasal congestion, and fever. There was no dyspnea, but dysgeusia and anosmia developed on the second day of admission. There were no comorbidities such as obesity or underlying pulmonary disease. The patient was found to have received the first and second doses of CoronaVac 19 days and 5 days prior to the onset of symptoms, respectively. Physical examination revealed normal vital signs, and lung examination demonstrated coarse rales, dominant in the lower left lung. Upon admission, the patient had a hemoglobin level of 14.8 g/dl, leukocyte count of 7.010/*µ*l, and platelet count of 258.000/*µ*l. The patient had a D-dimer level of 210 *µ*g/l and fibrinogen level of 271 mg/dl, with CRP and procalcitonin levels of 115 mg/l and 0.04 ng/ml, respectively. Laboratory examinations revealed SARS-CoV-2 PCR positive with RdRP Cq of 17.70, E-gene Cq of 17.27, and N-gene Cq of 16.65. Chest X-ray revealed a typical COVID-19 radiological appearance of diffuse infiltrate at the base which extended to the periphery of the lungs.

**Figure 1:**
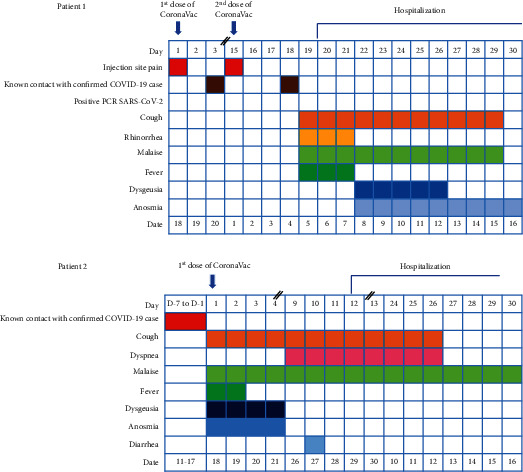
The history of vaccinations and development of symptoms in patient 1 after 1 dose of CoronaVac vaccine and in patient 2 after 2 doses of CoronaVac vaccine. In patient 1, two vaccination doses were completed 18 days before the first appearance of symptoms. In patient 2, the patient was vaccinated on the day the symptoms appeared. In both patients, exposure to confirmed COVID-19 cases occurred within 2 weeks prior to the presentation of symptoms.

**Figure 2:**
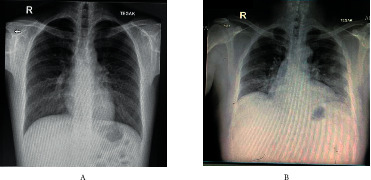
Chest X-rays of the patients. (a) Chest X-ray revealed a typical COVID-19 radiological appearance of diffuse infiltrate at the base, which extended to the periphery of the lungs. (b) Chest X-ray revealed a typical COVID-19 radiological appearance of basal nodular infiltrate at the periphery of the lungs, especially dominant in the left side.

The second patient, a 49-year-old man, came with the chief complaint of fever. The fever was present for 3 days and had started 11 days before admission, ranging from 37.7–38.0°C. The fever started on the same day when the patient received his vaccination. There was unproductive cough, and anosmia and dysgeusia developed on the same day with the fever onset. Diarrhea occurred 1 day before presentation to the hospital. The patient also had longstanding hypertension and diabetes mellitus without regular checkups or medications. At presentation, the patient was hypertensive, with a blood pressure of 190/80 mmHg. There were no rales or other physical abnormalities upon physical examination. Laboratory examination revealed hyperglycemia, hyponatremia hypoosmolar euvolemia, and hypokalemia. Procalcitonin was 0.08 ng/ml and CRP was 79.2 mg/l, while fibrinogen and D-dimer levels were 678.4 mg/dl and 380 *µ*g/l, respectively. Chest X-ray revealed a typical COVID-19 radiological appearance of basal nodular infiltrate at the periphery of the lungs, especially on the left side. Electrocardiography (ECG) revealed a complete right bundle branch block (RBBB). SARS-CoV-2 PCR on the admission day was positive with RdRP Cq of 28.27, E-gene Cq of 27.83, and N-gene Cq of 27.28. The timeline of vaccination and disease manifestation of both patients and chest X-ray are presented in Figures 1 and 2 respectively. Both patients had almost similar levels of S-RBD protein titers of 18 and 15 U/ml. Complete laboratory data of the patients can be found in Supplemental Data 1.

## 3. Discussion

We report two hospitalized patients with confirmed COVID-19 pneumonia and a history of CoronaVac vaccinations. The first patient with a relatively milder course of the disease had received two doses of CoronaVac, whereas the second patient with a more progressive course of the disease received only one dose before developing symptoms and being admitted to the hospital. Upon admission of the patients, we were presented with the scientific questions as follows: (1) whether the SARS-COV-2 infection occurred before the production of adequate neutralizing antibody by the vaccine, (2) whether the natural infection that occurred in the patients with prior vaccination did not boost the immune system, but instead trigger a severe manifestation of COVID-19, and (3) does the compatibility between the inactivated virus in the vaccine and the circulating strains of virus matter in determining vaccine efficacy.

With regard to exposure, it is highly likely that the patients encountered SARS-CoV-2 prior to either the first or second dose of vaccination in the case of the first patient or prior to the first dose in the second patient. It is therefore intriguing to speculate on the interaction between vaccination and the presence of active SARS-CoV-2 in an asymptomatic individual with low-level, high-level, or without preexisting neutralizing antibody. Both patients in this report did not have high levels of the RDP antibody.

**Figure 3:**
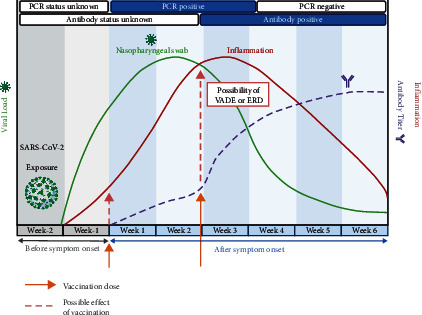
A schematic timeline of events showing SARS-CoV-2 exposure, PCR status, vaccine administration, the development of symptoms, inflammation, and the possibility of vaccine-associated disease enhancement (VADE) and enhanced respiratory disease (ERD). Upon the exposure to SARS-CoV-2, inflammation (red line) can occur until the symptom onset. During this period, the inflammation response can be amplified by the presence of VADE or ERD (pink arrow) when the patient had already been exposed to vaccinations (orange arrow).

**Figure 4:**
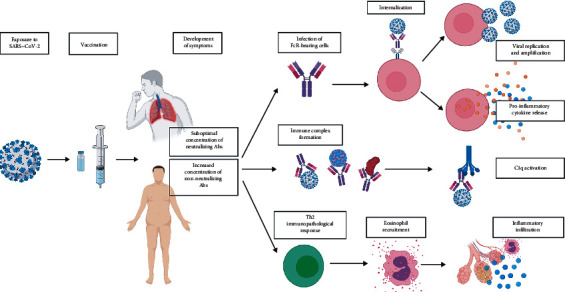
In VADE, the exposure to SARS-CoV-2 leads to asymptomatic infection, which is subsequently followed by vaccination and the development of symptoms. Patients then develop a suboptimal level of neutralizing antibody or increased level of nonneutralizing antibody or both. These lead to the development of VADE or ERD, in which immune response and inflammation are enhanced by the vaccine in previously infected individuals through the abovementioned mechanisms. Abs, antibodies.

Our observations suggest that vaccinations could act in boosting the inflammatory process and reveal the previously asymptomatic COVID-19 illness (Figure 3). COVID-19 signs and symptoms can manifest from the burden of viral replication and amplification, the activation of immune cells followed by the release of proinflammatory cytokines, or the formation of the immune complex (Figure 4) [6, 7]. We speculate that the exposure of these individuals to homotypic or heterotypic serotype viral infection and vaccine administration renders them susceptible to increased proinflammatory responses, leading to the exaggeration of symptoms.

Theoretically, vaccines could induce vaccine-associated disease enhancement (VADE), where only suboptimal, nonprotective titers of neutralizing antibodies were produced or proinflammatory T-helper type 2 response was induced. Secondly, enhanced respiratory disease (ERD) could manifest, where pulmonary symptoms are more severe due to peribronchial monocytic and eosinophilic infiltration [8–10]. Precise mechanisms underlying VADE have not been fully elucidated, but it is thought to occur in infections where cells with Fc receptors are targets of active viral infection and replication. SARS-CoV-2-positive monocytes and B and CD4 T lymphocytes had been detected in postmortem lung tissues. It is also thought that vaccines for viral infections with a high proinflammatory burden, such as RSV and SARS-CoV, are likely to result in VADE. Studies with inactivated SARS-CoV and respiratory syncytial virus vaccines have reported VADE via a Th2 cell response and lung eosinophilic infiltration, which may be worsened in aged hosts [8]. SARS-CoV-2 severity is primarily thought of as a result of cytokine storm [6], and dexamethasone was found to lower 28-day mortality in severe patients receiving oxygen supplementation [11]. Furthermore, antigens that induce nonneutralizing antibodies or insufficient neutralizing antibodies are likely to cause VADE [8]. *In vitro* experiments demonstrated the enhancement of SARS-CoV-2 entry into cells mediated with spike protein-specific antibodies in plasma from recovered elderly patients [12]. CoronaVac was reported to not have any observable antibody-dependent enhancement (ADE) of infection in macaques after SARS-CoV-2 challenge [13]; however, data from human trials are not currently available.

Our two cases suggest that determining whether vaccine recipients are SARS-CoV-2 infected albeit being asymptomatic could be important as vaccine administration could theoretically enhance inflammation and disease progression. VADE adds to the complexities of the sociodemographic and health factors as well as the extreme heterogeneity of individuals and populations [14]. VADE could also greatly affect both the decision-making process by individuals to get the vaccine as well as the decision-making process by governments and policymakers to choose certain vaccines and target certain populations with the limited vaccine doses available. It is therefore imperative to fully determine the risk, burden, and mitigation of VADE in order to increase the success of vaccine uptake [15].

When an individual is found to be PCR positive for SARS-CoV-2, we recommend checking antibody levels prior to vaccination for assessing whether the neutralizing antibody had been sufficiently produced as it is suggested that neutralizing antibodies only mediate ADE at the suboptimal neutralizing concentration [12]. Alternatively, levels of neutralizing antibody titer should be checked regardless of infection status prior to vaccination. Nonetheless, there is currently a lack of knowledge on the levels of baseline antibody titers and the minimum level of neutralizing antibodies that would confer protection against COVID-19. Studies reported that neutralizing antibodies were detected starting from day 10 and peaked at weeks 3 (IgG) and 4 (IgM) [16]. In our patients, vaccinations were performed in less than 3 weeks before the measurement of antibody titers, further strengthening the notion that neutralizing antibodies had not yet been formed optimally, which posed the risk of VADE or ERD. Furthermore, vaccines with a high theoretical risk of inducing VADE or ERD include inactivated viral vaccines, such as CoronaVac, which was given to our patients, which could involve nonneutralizing antigen targets and/or the S protein in nonneutralizing conformations, further providing a range of nonprotective targets for antibodies that could drive additional inflammation [17].

In conclusion, we report two cases of patients developing COVID-19 shortly after vaccination with CoronaVac in which VADE is highly likely. We are currently investigating the relationship between inactivated virus strains in the vaccine and the circulating strains of virus in determining vaccine efficacy. As an increasing number of individuals are being vaccinated, more data are needed on VADE. To date, no clinical markers are in use to distinguish between naturally progressive, severe COVID-19 and VADE in clinical practice. We recommend that current vaccination strategies consider the measurement of neutralizing antibody titers as a guide in ensuring the safest strategy for mass immunization. Fully determining the risk, burden, and mitigation of VADE is warranted in order to increase the success of vaccine uptake. Further studies are needed to investigate the true incidence of VADE on vaccinated individuals as well as on how to differentiate between VADE and severe manifestations of COVID-19 that are unrelated to vaccination.

## Data Availability

All data are provided in Supplemental Data 1.
