# Supplementary material for: Vaccine-Associated Disease Enhancement (VADE): Considerations in Postvaccination COVID-19
Source: Case Rep Med. 2021 Oct 29;2021:9673453. doi: 10.1155/2021/9673453 (PMC8570879; doi:10.1155/2021/9673453)
Supplement: Supplementary Materials — Laboratory data of the patients are available in Supplemental Data 1. [file 9673453.f1.docx]

**Supplementary Data 1**

Laboratory Values

| Normal range | Patient 1, 6/2/2021 | Patient 2, 8/2/2021 |
| --- | --- | --- |
| Hemoglobin (13-17 g/dl) | 14,8 | 14,2 |
| Hematocrite (40-50%) | 44,3 | 38 |
| Leukocyte (4-10x10^3^ /ul) | 7.010 | 9.180 |
| Thrombocyte (150-410x10^3^ /ul) | 258.000 | 228.000 |
| D-dimer (<400ug/L) | 290 | 380 |
| CRP (0.7 mg/L) | 0,7 | 79,2 |
| SGOT/SGPT (5-34 U/L; 0-55 U/L)) | 24/29 | 34/49 |
| Ureum (19-44 mg/dl) | 20 | 29,4 |
| Creatinine (0,73-1,18 mg/dl) | 0,9 | 1,0 |
| eGFR-EPI (mL/min/1.73m^2^) | 119,1 | 88 |
| Random plasma glucose (60-140 mg/dl) | 73 | 157 |
| Natrium (136-145 mEq/L) | 136 | 134 |
| Kalium (3,5-5,1 mEq/L) | 4 | 2,8 |
| Chloride (98-107 mEq/L) | 103,5 | 92 |
| PT (10,9-11,7 seconds) | 10,9 | 10,5 |
| APTT (31-32,8 seconds) | 31 | 32,8 |
| Fibrinogen (200-400 mg/dl) | 271 | 678 |
| Procalcitonin (<0,05 ng/ml) | 0,04 | 0,08 |
